# Observations on Angina Pectoris

**Published:** 1829-01-01

**Authors:** 


### XXIII.

### ANGINA PECTORIS.

A case of this disease, caused, it is said, by "chronic inflammation and partial ossification of the coronary and *vertebral* arteries," is briefly related in Dr. Farre's semi-annual Journal, No. I.

A baker by trade, aged 57, died suddenly on the 16th of November, 1826. For some years previously he had been subject to attacks of extreme dyspnœa, pain in the chest, and *violent cough.* These symptoms came on suddenly, while engaged in exercise, especially in ascending stairs, or any other muscular exertion which accelerated the respiration. He termed his disease asthma, as is usually the case. He could not endure the horizontal posture, and always reclined with his shoulders raised. His death was instantaneous, and without premonition.

*Dissection.* The ventricles of the brain contained four or five ounces of water—vertebral arteries unequally enlarged, and partially ossified. Abdominal viscera were sound. The lungs were healthy, but gorged, even to blackness, with blood. There were two ounces of fluid in the pericardium. The coronary arteries were inflamed, and partially ossified. No account is given of the state of the muscular structure of the organ, or of the nerves leading to it.

In a commentary on this case, Dr. Farre passes the following judgment. "The death, in the Editor's opinion, is to be referred *exclusively*, to a paroxysm of *angina pectoris*, resulting from the partially *ossified* state of the *coronary* arteries." We find ourselves obliged to dissent from this opinion in two important points. In the first place, the case is by no means an unequivocal instance of angina pectoris. Excepting the solitary symptom of dyspnœa, it did not exhibit a single phenomenon of the disease in question—and for the truth of this remark, we refer to the written descriptions of angina pectoris, especially to the work of Dr. Parry—but above all, to the experience and observation of any and every practitioner who has ever witnessed the disease. Mr. Gore, who communicated the case to Dr. Farre, does not appear to have seen the patient before his death, and only collected the foregoing particulars from the friends of the deceased! Where is the dreadful stricture across the chest—the sense of dying—the agonising pain darting along one of the arms—the pallid countenance, and many other characteristics of angina pectoris? Is "*violent cough*" a symptom of the disease under consideration? Dr. Farre has headed the case as one of angina pectoris, *produced* by "chronic inflammation and partial ossification of the coronary and *vertebral* arteries;" though, in his Commentary, he attributes the disease, and consequently, the death of the patient, to partial ossification of the coronary artery exclusively! We are very much surprised that such a man as Dr. Farre should cling to an untenable and antiquated doctrine, after all the lights which have been thrown on diseases of the heart by modern pathological researches. *Five ounces* of water in the ventricles of the brain—*enlarged* and ossified vertebral arteries—lungs "nearly black," with engorgement—all these things are passed over as a feather in the balance, while a few specks of ossification in the coronary artery of the heart, are looked upon as the cause of death! No mention is made of the muscular structure of the heart—no examination of the nerves!

That the disease termed angina pectoris may depend on more than one pathological condition of the heart cannot now be doubted; but, that it is, in the great majority of instances, a neuralgic affection of the organ, we firmly believe. In most of the cases which we have had an opportunity of examining, the muscular structure was flabby and wasted—or loaded with fat. What do we see in neuralgic affections elsewhere? Wasting and weakness of the muscles. Death, in angina pectoris, we believe to result from one of two conditions—spasm, or momentary paralysis of the organ. From spasm, when the patient dies in agonies, as is sometimes the case—from momentary paralysis, when he dies instantaneously, without suffering. In the latter case, it is of *syncope*, in fact, which the individual expires—and the disease, indeed, has been denominated "*syncope* anginosa." Why should we wonder that the heart may be affected with temporary paralysis, as well as other muscles? There is scarcely a muscle in the body, voluntary or involuntary, that has not been found to be the seat of temporary paralysis. This temporary loss of power in the heart must, of course, be fatal, if it last beyond a certain time. In common syncope, from emotions of the mind, loss of blood, or other causes, power is restored before the vital spark is fled—though not always, for death sometimes takes place, even from this momentary cessation of action in the central organ of the circulation. It is hardly necessary to say, that to attribute the disease, in the case above narrated, to the specks of ossification on the coronary arteries, when other, and far more serious lesions, were discovered, (though perhaps still more lay behind) on dissection, is a sad specimen of philosophising in medicine! We would recommend to those young aspirants for professional fame, who are, as yet, unencumbered with the harrassing duties of private practice, to direct their attention to the pathology of the visceral nerves. We cannot, indeed, expect to discover the causes of functional disorder in the nervous system so readily as in the arterial system; but still we believe there is much appreciable lesion to be detected by accurate dissection. In the mean time, we cannot but deplore the rage for tracing disorders of the human machine entirely to physical alterations of structure, knowing, as we do, that all diseases, or almost all, originate in disordered function, connected with, if not entirely dependent on, the nervous system. According, indeed, to some modern medical philosophers, the brain and nerves are almost useless parts of the animal machine—while the mechanical or physical agencies are every thing. The laws of hydraulics and chemistry are soon to supersede the laws of vitality—and the *morale* is soon to be entirely absorbed in the *physique.*

